# Construction of Multilevel Structure for Avian Influenza Virus System Based on Granular Computing

**DOI:** 10.1155/2017/5404180

**Published:** 2017-01-16

**Authors:** Yang Li, Qi-Hao Liang, Meng-Meng Sun, Xu-Qing Tang, Ping Zhu

**Affiliations:** School of Science, Jiangnan University, Wuxi 214122, China

## Abstract

Exploring the genetic structure of influenza viruses attracts the attention in the field of molecular ecology and medical genetics, whose epidemics cause morbidity and mortality worldwide. The rapid variations in RNA strand and changes of protein structure of the virus result in low-accuracy subtyping identification and make it difficult to develop effective drugs and vaccine. This paper constructs the evolutionary structure of avian influenza virus system considering both hemagglutinin and neuraminidase protein fragments. An optimization model was established to determine the rational granularity of the virus system for exploring the intrinsic relationship among the subtypes based on the fuzzy hierarchical evaluation index. Thus, an algorithm was presented to extract the rational structure. Furthermore, to reduce the systematic and computational complexity, the granular signatures of virus system were identified based on the coarse-grained idea and then its performance was evaluated through a designed classifier. The results showed that the obtained virus signatures could approximate and reflect the whole avian influenza virus system, indicating that the proposed method could identify the effective virus signatures. Once a new molecular virus is detected, it is efficient to identify the homologous virus hierarchically.

## 1. Introduction

Exploring the genetic structure of biological population attracts the focus in the field of population biology, molecular ecology, and medical genetics [1]. Influenza A virus is a negative-strand RNA virus, which encodes the 8 structural proteins and 2 nonstructural proteins. In the past several decades, some subtypes of influenza viruses have been identified to infect humans, whose epidemics cause morbidity and mortality worldwide [2, 3]. Subtyping identification of a virus is typically based on viral hemagglutinin (HA) and neuraminidase (NA) fragments among the 10 encoded proteins [4, 5]. So far, dozens of subtypes, combination of the 16 HA and 9 NA types, make up the whole viral system and it was verified that different labeled viruses descend from the same ancestor according to microscopic structural features and genome organization analysis [6]. Evolutionary forces, treated as the most important molecular mechanisms, such as natural selection acting upon rapidly mutating viral populations could shape the genetic structure of influenza viruses in different hosts, geographic regions, and periods of time with genetic mutation [7]. In addition, influenza viruses are equipped with antigenic changes, known as antigenic shifts among different subtypes of influenza viruses, which results in structural changes to escape the immunity [8]. It is of crucial importance to identify the subtypes and analyze the evolutionary relationships for developing antiviral drugs and vaccines. Thus, accessing the viral genomes in a timely fashion and developing effective analyzing methods are urgently needed.

The dramatic progress in sequencing technologies provides unprecedented prospects for the exploration of virus homologous and mutation trajectory in space and time. Understanding the evolution of influenza viruses has benefited from phylogenetic reconstructions of the hemagglutinin protein [9]. In an alternative approach, Lapedes and Farber [10] applied a technique called multidimensional scaling to study antigenic evolution of influenza. Plotkin et al. [8] clustered hemagglutinin protein sequences using the single-linkage clustering algorithm and found that influenza viruses group into several clusters. Upon the dimensional projection technique to characterize hemagglutination inhibition (HI) data, a low-dimensional clustering method that can detect the clusters containing an incipient dominant strain was presented by He and Deem [11]. However, those works just focused on the one fragment, especially HA protein, to explore the evolutional relationships. And large volume of data poses some daunting challenges for exploring the structure of the complex system and the intrinsic relationship. Therefore, there is a need for less computationally intensive methods.

In recent years, the granular computing (GrC) theory has become a hotspot in the field of artificial intelligence and machine learning, which comes from the idea that people solve the problems from different levels and views [12]. Clustering technique is an effective way to generate granules of complex system. Y. Y. Yao and J. T. Yao accomplished a series of research work for applying the theory to data mining and some other fields [13]. Hartmann et al. [14] proposed supervised hierarchical clustering in fuzzy model identification by using hierarchical tree construction. Tang et al. [15, 16] introduced the granular space to describe the hierarchical structural information by using the algebraic topology based on the fuzzy quotient space theory [12]. He also studied the hierarchical clustering structure and analyzed the fuzzy equivalence (or proximity) relation based on the fuzzy granular space. Constructing the hierarchical structure of complex system and extracting the essential information among the granules on different granularities are the goals.

In this paper, our aim is to explore the evolutional relationships of the avian influenza viruses in the same subtype and among the subtypes considering both HA and NA fragments in the virus system. Moreover, the complex virus system should be reduced for further exploration, faced with thousands of samples in the dataset. Jointing the two protein sequences, the feature vectors are extracted from HA and NA proteins, respectively, for labeling the specific virus. Furthermore, the granular signatures in the viral granules are identified based on the obtained features to reduce the systematic and computational complexity and then its performance will be evaluated. This will provide the supports for the rationality of subtype identification. Once a new molecular virus is detected, it could be analyzed with obtained viral signatures and then the prevention and treatment measures can follow what were applied in the viral signature.

## 2. Materials and Methods

### 2.1. Materials

The influenza virus dataset was downloaded from the NCBI Influenza Virus Resource (http://www.ncbi.nlm.nih.gov/genomes/FLU/) [17]. The influenza virus contains eight linear negative-strand RNA fragments, which encode 10 viral proteins, that is, PB1, PB2, PA, HA, NP, NA, M1, M2, NS1, and NS2, among which most are structural proteins except NS1 and NS2. Notably, HA and NA fragments play the direct and important roles in the viral subtyping identification and the functions [18]. It has been verified that 8 subgroups of avian influenza virus (H5N1, H5N2, H7N2, H7N3, H7N7, H9N2, H10N7, and H7N9) could infect people, which occurred from 1902 to 2015 around the world. The avian influenza viruses are labeled with unambiguous symbols such as the host, outbreak time, and detection sites. Removing some vague and uncompleted viruses, there are 8274 influenza viruses which reserve HA and NA protein fragments simultaneously (13143 HA protein fragments and 9401 NA protein fragments), compositing the whole avian virus protein system, denoted as *Ω*. According to the physicochemical property [19], amino acids are divided into four types, namely, the polar and hydrophilic (*pq*), polar and hydrophobic (*pr*), nonpolar and hydrophilic (*sq*), and nonpolar and hydrophobic (*sr*). Considering the adjacency statistical information, the 16-dimension feature vector is extracted by calculating the frequency from one protein sequence. Therefore, 32-dimension feature vector is extracted to represent a virus molecule.

### 2.2. The Optimization Model for Extracting the Hierarchical Structure

A relation *R* on a universe *X* is a fuzzy proximity (FP) relation if it satisfies the reflexivity and symmetry [16, 20]. Furthermore, if *R* is an FP relation on the universe *X* and satisfies the separable condition (∀*x*, *y* ∈ *X*, *R*(*x*, *y*) = 1↔*x* = *y*), then *R* is called a separable FP relation (or SFP relation).

In [16], the granular space of FP (or SFP) relations on the universe *X* was introduced, and then their properties were explored. Let *R* be an FP (or SFP) relation on a finite universe *X* = {*x*_1_, *x*_2_,…, *x*_*n*_}, where *X* is a dataset of *K*-dimension space. For any *λ* ∈ [0,1], we define a relation *R*_*λ*_ : (*x*, *y*) ∈ *R*_*λ*_↔*R*(*x*, *y*) ≥ *λ*, where *R*_*λ*_ is a crisp proximity relation that satisfies the reflexivity and symmetry. Then, the equivalent classes of the transitive closure tr(*R*_*λ*_) can be marked by [*x*]_*λ*_, which is derived by *R*_*λ*_, and then *X*(*λ*) = {[*x*]_*λ*_∣*x* ∈ *X*} is a granularity corresponding to *λ*. The set {*X*(*λ*)∣*λ* ∈ [0,1]} represents a fuzzy granular space on *X*, which is an ordered set, and satisfies that the bigger the threshold *λ* is, the finer the granularity is, denoted by *ℵ*_TR_(*X*) [16].

The granularity derived by *λ* is marked as *X*(*λ*) = {*a*_1_, *a*_2_,…, *a*_*c*_*λ*__}, where *a*_*i*_ = {*x*_*i*1_, *x*_*i*2_,…, *x*_*iJ*_*i*__} satisfying the conditions that |*a*_*i*_| = *J*_*i*_ (|·| stands for the number of the elements in a set) and ∑_*i*=1_^*c*_*λ*_^*J*_*i*_ = *n*. Some properties are explored, such as $\bar{a_{i}}=\sum_{k=1}^{J_{i}}x_{ik}/J_{i}$ (*i* = 1,2,…, *c*_*λ*_) is the center of granule *a*_*i*_ and the center of *X* is $\overset{-}{a}=\sum_{i=1}^{c_{\lambda }}\sum_{k=1}^{J_{i}}x_{ik}/n$. From the perspective of statistical theory, two indexes are introduced to measure the deviations within the classes and among the classes on the granulation *X*(*λ*) [18, 20], defined, respectively, as follows:

(1) SamongXλ=∑i=1cλJiai¯−a¯22n,SwithinXλ=∑i=1cλ∑k=1Jixik−a¯22n,

where ‖·‖_2_ stand for the 2-norm number in *K*-dimension space.

By analyzing the variance within and among the classes in statistics [21], *S*_among_(*X*(*λ*)) is monotone increasing, with the granularity changing from the coarse to the fine, while *S*_within_(*X*(*λ*)) is gradually decreasing. Notably, the total deviation (*S*(*X*(*λ*)) = *S*_among_(*X*(*λ*)) + *S*_within_(*X*(*λ*))) is always constant $S\left(X(\lambda )\right)=\sum_{i=1}^{n}{\left\|x_{i}-\bar{a}\right\|}_{2}^{2}/n$. Additionally, *S*_among_(*X*(0)) = *S*_within_(*X*(1)) = 0 and $S_{\text{among}}\left(X(1)\right)=S_{\text{within}}\left(X(0)\right)=\sum_{i=1}^{n}{\left\|x_{i}-\bar{a}\right\|}_{2}^{2}/n$. Therefore, a fuzzy hierarchical evaluation index (FHEI) based on the fuzzy granular space is proposed as follows:

(2) $$\begin{array}{c} \text{FHEI}\left(\;X\left(\;\lambda \;\right)\;\right)=\left|\;S_{\text{among}}\left(\;X\left(\;\lambda \;\right)\;\right)-S_{\text{within}}\left(\;X\left(\;\lambda \;\right)\;\right)\;\right|. \end{array}$$

We establish an optimization model to determine the reasonable granulation in the granular space with the minimal objective; that is, FHEI(*X*(*λ*)) reaches the minimum. There exists only one *λ* = *λ*_0_ to meet the optimization model, marked as Model (2):

(3) $$\begin{array}{c} X\left(\;\lambda_{0}\;\right)=arg\underset{X\left(\lambda \right)\in {ℵ}_{\text{TR}}\left(X\right)}{\min}\left\{\;FHEI\left(\;X\left(\;\lambda \;\right)\;\right)\;\right\}. \end{array}$$

**Remark 1 .**

Model (2) is a global optimization model without constraints on the hierarchical structure of the finite universe *X*. Compared with [18], their model for determining the optimal hierarchical clustering has the restriction *S*_among_(*X*(*λ*)) > *S*_within_(*X*(*λ*)).

Given an FP relation (or SFP relation) *R* on the finite set *X* = {*x*_1_, *x*_2_,…, *x*_*n*_} and *D* = {*R*(*x*, *y*)∣*x*, *y* ∈ *X*} = {*r*_0_, *r*_1_,…, *r*_*N*_}, satisfying 1 = *r*_0_ > *r*_1_ > ⋯>*r*_*N*_, an algorithm is presented to detect the optimized hierarchical clustering and construct the hierarchy of complex system based on the fuzzy granular space [16].

**Algorithm 2.2 A.**

 

Input: an FP relation (or SFP relation).

Output: the optimized hierarchical structure and the corresponding threshold.

*Step 1*

(4) Xri=C=a1,a2,…,ac1,S0⟸SamongXri−SwithinXri i=0.

*Step 2*

(5) $$\begin{array}{c} i⟸i+1. \end{array}$$

*Step 3*

(6) $$\begin{array}{c} A⟸C. \end{array}$$

*Step 4*

(7) B⟸⌀,C⟸⌀.

*Step 5*. For any *a*_*j*_ ∈ *A*, *B* ⇐ *B* ∪ *a*_*j*_, *A* ⇐ *A*∖*a*_*j*_.

*Step 6*. For ∀*a*_*k*_ ∈ *A*, if ∃*x*_*j*_ ∈ *a*_*j*_, *y*_*k*_ ∈ *a*_*k*_ satisfying *R*(*x*_*j*_, *x*_*k*_) ≥ *r*_*i*_, *B* ⇐ *B* ∪ *a*_*k*_, *A* ⇐ *A*∖*a*_*k*_.

*Step 7*

(8) $$\begin{array}{c} C⟸\left\{\;B\;\right\}\cup C. \end{array}$$

*Step 8*. If *A* = *⌀*, *X*(*r*_*i*_) = *C*; otherwise, go to Step 5.

*Step 9*. If *X*(*r*_*i*_) ≠ *X*(*r*_*i*−1_),  *S*_1_ ⇐ |*S*_among_(*X*(*r*_*i*_)) − *S*_within_(*X*(*r*_*i*_))|; otherwise, go to Step 2.

*Step 10*. If *S*_0_ > *S*_1_, *S*_0_ ⇐ *S*_1_, go to Step 2.

*Step 11*. Output *r*_*i*−1_, *X*(*r*_*i*−1_) and *S*_0_.

The computational complexity of Algorithm A is *O*(*n*^2^). The concrete problems are decomposed hierarchically, which is consistent with the core idea of GrC. Given an FP (or SFP) relation on the finite set *X*, the optimization clustering structure constructed by Algorithm A is its first level structure. Furthermore, its second level structure is obtained if Algorithm A is repeatedly applied to all the equivalent classes in its first level structure. Therefore, Algorithm A can be used to construct multilevel structure in practical application.

### 2.3. Identification of Granular Signature

Once the optimal granularity of the complex system is determined, it is of crucial importance to construct information granules for abstracting original samples. Generally, the granules are obtained according to the principle: the samples with the same features assemble in one granule. And the average of all samples in one class or the center of the class is efficacious to represent the core information. Suppose that a multilevel structure (or granularity) *X*^*∗*^ = {*a*_1_, *a*_2_,…, *a*_*J*_} is constructed, where *J* = |*X*^*∗*^|. To reduce the complexity of the system, feature viruses (or signature viruses) could be extracted to approximately represent the equivalent class. According to the nearest-to-center principle, an objective function to select the signature is established, and it is formulated as follows:

(9) $$\begin{array}{c} p_{i}=\arg\underset{1\leq k\leq J_{i}}{\max}\left\{\;R\left(\;x_{ik},\bar{a_{i}}\;\right)\;\right\}, \end{array}$$

where *p*_*i*_ is the signature item of the granule *a*_*i*_ and *P* = {*p*_1_, *p*_2_,…, *p*_*J*_} is a signature set of the granularity *X*^*∗*^. In some way, the signature set *P* can be used to represent approximately the complex system *X*.

### 2.4. Validation of Granular Signature Set

To evaluate the performance of selected signature set *P*, a classifier is designed for classifying the rest of the samples of the corresponding classes according to the principle of maximum similarity, marked as Model (3). Given a virus *q*_*j*_ (∈*X*∖*P*), the classifier is designed:

(10) $$\begin{array}{c} L_{j}=\arg\underset{i}{\max}\left\{\;R\left(\;q_{j},p_{i}\;\right)\;\right\}, \end{array}$$

where *p*_*i*_ ∈ *P*, *i* = 1,2,…, *J*, and *L*_*j*_ is the class the virus *q*_*j*_ belongs to.

Model (3) states that the signature viruses are treated as the classifying targets and the other samples in *X*∖*P* are assigned to |*P*| classes. All samples in *X*∖*P* are divided into |*P*| classes according to Model (3), marked as *b*_*k*_,  *k* = 1,2,…, |*P*|. The accuracy ratio *r* is introduced to measure the efficiency of signature set for constructing the multilevel structure *X*^*∗*^. It is defined as

(11) $$\begin{array}{c} r=\frac{\sum_{k=1}^{\left|P\right|}\left|a_{k}\cap b_{k}\right|}{\left|X\setminus P\right|}. \end{array}$$

In formula (11), the overlapped ratio *r* is proposed, which measures the rationality of the obtained signature to represent the whole virus system. And the bigger the value *r* is, the better the result is.

## 3. Results and Analysis

In this section, we apply the proposed model to the avian influenza virus system for constructing the evolutionary structure, which contains 8274 viral HA and NA protein fragments simultaneously within 8 subtypes, listed in Table 1.

Based on the feature vectors extracted from the viral HA and NA proteins, the 32-dimension vector *x*_*i*_ = (*x*_*i*_1__, *x*_*i*_2__,…, *x*_*i*_32__) labels the specific virus *x*_*i*_. Furthermore, the similarity between viruses *x*_*i*_ and *x*_*j*_ is measured:

(12) $$\begin{array}{c} R\left(\;x_{i},x_{j}\;\right)=\frac{\left(x_{i},x_{j}\right)}{\sqrt{\left(x_{i},x_{i}\right)\cdot \left(x_{j},x_{j}\right)}}, \end{array}$$

where (*x*_*i*_, *x*_*j*_) = ∑_*i*=1_^32^*x*_*ik*_ · *x*_*jk*_ stands for the inner product in 32-dimension space. Obviously, *R* is an SFP relation.

The virus dataset has redundant information as many viruses are labeled with the same host, the same occurrence time, and the same outbreak sites, which could pose the obstacle to explore the intrinsic relationship and difference among the subtypes. Thus, those with the same host, the same occurrence time, and the same location combine as one new point (a representative virus), which is the preliminary system simplification, and then a unique virus database *Ω*^*∗*^ is obtained. The FHEI is applied to virus system *Ω*^*∗*^ containing 909 avian influenza viruses, to obtain the reasonable partition and evolutionary structure.

On the basis of the virus database *Ω*^*∗*^, the viral granular space (evolutionary structure) is constructed by using Algorithm A. On the first level, 3 equivalent classes were finally determined to partition the whole system, and the corresponding signature viruses are obtained, shown in Table 2. For the virus granules on the first level, class A1 contains the most viruses (about 93.5%), and granule A3 arises, containing an isolated virus (A/American green-winged teal/Washington/1595750/2014(H5N1)). Therefore, it is necessary to construct the second level of virus system. For each virus granule on the first level, Algorithm A is used repeatedly, which is to refine the granules to get the detailed evolutional structure. 14 equivalent subclasses are identified, denoted as *b*_*k*_^*∗*^ (*k* = 1,2,…, 14), and the virus signatures are extracted, shown in Table 3. From Tables 2 and 3, we construct the two-level feature structure of the whole virus system by using the signature viruses on first level and second level structure.

The virus signature could be used to approximate the whole system for they are selected from the classes as the granule information. Moreover, the classifier, designed based on the principle of maximum similarity, is applied to validate the performance of virus signature. The accuracy rate of the signature virus set *P*^*∗*^ on the second level structure is 76.57% by comparison, indicating that the second level structure of viruses system constructed by our model is effective.

**Remark 2 .**

Evaluating the performance of virus signature, the error rate is still 23.43%, which might be caused by the approximation process since all signature viruses are selected according to the nearest-to-center principle and they are not just on the center of each subclass, respectively. From the perspective of approximation, the signature set contains the most information of virus system according to the accuracy rate 76.57%. Therefore, the signature virus set *P*^*∗*^ containing 14 viruses can be used to approximate the whole system containing 909 viruses.

**Figure 1:**
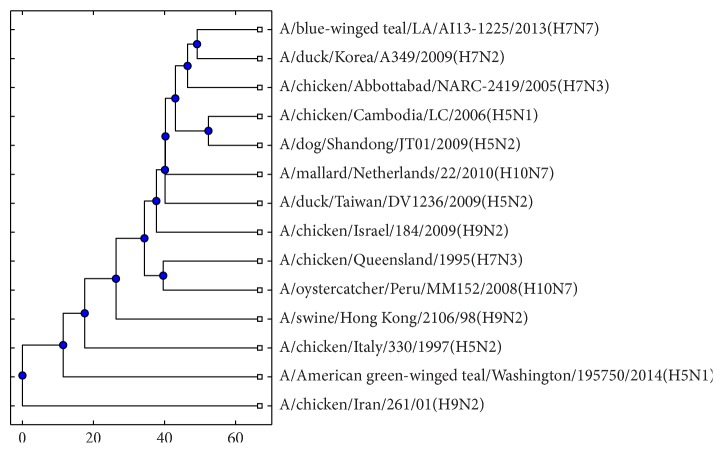
The phylogenetic tree of signature viruses on second level structure.

The phylogenetic tree of the signature virus set *P*^*∗*^ can be constructed by applying the hierarchical clustering algorithm [16], shown in Figure 1. According to Remark 2, it can also be treated as the core structure of whole influenza viruses system, which helps us understand the evolutionary history and the mechanism of evolution [22].

Among the 8 virus subtypes, 7 viruses are identified as the signature viruses except H7N9, for H7N9 viruses account for the minority of whole system (Figure 1). Exploring the intrinsic relation, it is obvious that H7N9 belongs to class B4, elucidating that the variation of H7N9 is not significant [23] and can be viewed as a new member in the family of viruses. Based on the coarse-grained idea, one signature virus represents the corresponding class. However, some isolated points are detected, such as A/chicken/Cambodia/LC/2006(H5N1), A/dog/Shandong/JT01/2009(H5N2), and A/chicken/Queensland/1995(H7N3), which might be caused by the big change to virus RNA strain.

From the hierarchical structure of the feature viruses, A/blue-winged teal/LA/AI13-1225/2013(H7N7), A/duck/Korea/A349/2009(H7N2), and A/chicken/Abbottabad/NARC-2419/2005(H7N3) have similar evolution relationship (connect closely) for they equip the same HA type (H7). Besides, A/chicken/Cambodia/LC/2006(H5N1) and A/dog/Shandong/JT01/2009(H5N2) have the consistent conclusions. However, A/chicken/Italy/330/1997(H5N2) is far from them, which could be due to the fact that the outbreak time plays an important role in sequence mutation. If just considering the HA and NA proteins, the subtypes, such as H9N2 [24], should be redefined. Comparing the two-level structure and hierarchical structure of virus signature, the intrinsic relationship among A1 on the first level structure is consistent with that in the hierarchical structure, while class A2 has the dispersed structure where the feature viruses in different subtypes scatter in chaos, which indicates that constructing the second level structure is meaningful.

## 4. Conclusions

The rapid variation of influenza viruses results in low-accuracy subtyping identification and makes it difficult to develop effective drugs. This article explored the homology of avian influenza virus system and identified the subtypes according to HA and NA protein fragments, which might provide the support for developing antiviral drugs and vaccines according to different subtypes. Phylogenetic reconstructions serve understanding the evolution of influenza viruses. However, the large amounts of virus dataset pose an obstacle for analyzing the evolutionary relationship and identifying the correct subtypes to predict the biological functions. Granular computing theory was applied to determine the partition of virus system based on the constructed granular space. A method and the corresponding algorithm were proposed for detecting the rational granularity. With the proposed algorithm applied repeatedly, a multilevel structure of whole system was constructed. To reduce the computational complexity, some key viruses were selected to approximate the whole system based on the coarse-grained idea. According to the nearest-center principle, virus signatures were identified and constructed the granular signature set of a multilevel structure of complex system. By designing a classifier, the performance of virus signatures was evaluated and the result showed that the virus signatures could reflect the most properties of virus system. Furthermore, hierarchical structure of virus signature was constructed by using hierarchical clustering algorithm. Both of the two structures have some consistent intrinsic relationship among the virus systems and between the different subtypes. Some viruses were detected as isolated points in the structure thought equipped with the same labels, which might be caused by the rapid variations in the RNA strands. The virus signatures have the potential use in new virus subtyping comparison and functional prediction.

## Figures and Tables

**Table 1:** The 8 subtypes of avian influenza virus.

Subtype	Number	Subtype	Number	Subtype	Number	Subtype	Number
H5N1	306	H5N2	127	H7N3	70	H9N2	199
H7N9	24	H7N2	40	H7N7	68	H10N7	75

**Table 2:** Three signature viruses of the first level structure.

Number	Virus number	Virus signature
A1	850	A/Pekin duck/Singapore/F59/04/98(H5N2)
A2	58	A/chicken/Tunisia/145/2012(H9N2)
A3	1	A/American green-winged teal/Washington/1595750/2014(H5N1)
Sum	909	

**Table 3:** The virus classes on the second level structure.

Number	Virus number	First level	Virus signature
B1	1	A1	A/chicken/Cambodia/LC/2006(H5N1)
B2	1	A1	A/dog/Shandong/JT01/2009(H5N2)
B3	177	A1	A/chicken/Israel/184/2009(H9N2)
B4	665	A1	A/duck/Taiwan/DV1236/2009(H5N2)
B5	1	A1	A/blue-winged teal/LA/AI13-1225/2013(H7N7)
B6	2	A1	A/duck/Korea/A349/2009(H7N2)
B7	2	A1	A/chicken/Abbottabad/NARC-2419/2005(H7N3)
B8	1	A1	A/mallard/Netherlands/22/2010(H10N7)
B9	12	A2	A/swine/Hong Kong/2106/98(H9N2)
B10	35	A2	A/chicken/Italy/330/1997(H5N2)
B11	1	A2	A/chicken/Queensland/1995(H7N3)
B12	9	A2	A/chicken/Iran/261/01(H9N2)
B13	1	A2	A/oystercatcher/Peru/MM152/2008(H10N7)
B14	1	A3	A/American green-winged teal/Washington/195750/2014(H5N1)
